# Risk of bias: a simulation study of power to detect study-level moderator effects in meta-analysis

**DOI:** 10.1186/2046-4053-2-107

**Published:** 2013-11-28

**Authors:** Susanne Hempel, Jeremy NV Miles, Marika J Booth, Zhen Wang, Sally C Morton, Paul G Shekelle

**Affiliations:** 1 RAND Corporation, Santa Monica, CA 90407, USA; 2 Mayo Clinic, Rochester, MN 55905, USA; 3 University of Pittsburgh, Pittsburgh, PA 15261, USA; 4 Veterans Affairs Greater Los Angeles Healthcare System, North Hills, CA 91343, USA

**Keywords:** Meta-analysis, Power, Heterogeneity, Meta-epidemiological dataset, Randomized controlled trial (RCT)

## Abstract

**Background:**

There are both theoretical and empirical reasons to believe that design and execution factors are associated with bias in controlled trials. Statistically significant moderator effects, such as the effect of trial quality on treatment effect sizes, are rarely detected in individual meta-analyses, and evidence from meta-epidemiological datasets is inconsistent. The reasons for the disconnect between theory and empirical observation are unclear. The study objective was to explore the power to detect study level moderator effects in meta-analyses.

**Methods:**

We generated meta-analyses using Monte-Carlo simulations and investigated the effect of number of trials, trial sample size, moderator effect size, heterogeneity, and moderator distribution on power to detect moderator effects. The simulations provide a reference guide for investigators to estimate power when planning meta-regressions.

**Results:**

The power to detect moderator effects in meta-analyses, for example, effects of study quality on effect sizes, is largely determined by the degree of residual heterogeneity present in the dataset (noise not explained by the moderator). Larger trial sample sizes increase power only when residual heterogeneity is low. A large number of trials or low residual heterogeneity are necessary to detect effects. When the proportion of the moderator is not equal (for example, 25% ‘high quality’, 75% ‘low quality’ trials), power of 80% was rarely achieved in investigated scenarios. Application to an empirical meta-epidemiological dataset with substantial heterogeneity (I^2^ = 92%, τ^2^ = 0.285) estimated >200 trials are needed for a power of 80% to show a statistically significant result, even for a substantial moderator effect (0.2), and the number of trials with the less common feature (for example, few ‘high quality’ studies) affects power extensively.

**Conclusions:**

Although study characteristics, such as trial quality, may explain some proportion of heterogeneity across study results in meta-analyses, residual heterogeneity is a crucial factor in determining when associations between moderator variables and effect sizes can be statistically detected. Detecting moderator effects requires more powerful analyses than are employed in most published investigations; hence negative findings should not be considered evidence of a lack of effect, and investigations are not hypothesis-proving unless power calculations show sufficient ability to detect effects.

## Background

There are both theoretical and empirical reasons to believe that trial design and execution factors are associated with bias. Bias is the systematic deviation of an estimate from the true value. One example of a trial feature is randomization. Randomly allocating experimental subjects to control and intervention groups to ensure that groups are comparable at baseline was originally introduced in agricultural science and adopted by medical researchers [1]. The importance of several such features of what is now known as trial quality or risk of bias has been recognized for hundreds of years: the first published blinded (or masked) experiments with placebo controls were carried out in 1784 [2].

Critical appraisal of research studies is of particular importance to systematic reviews, which aim to summarize the available evidence adequately. Methodological characteristics of studies included in a systematic review can have a substantial impact on treatment effect estimates [3]. Heterogeneity describes the variance among study results and it is a function of random variation and systematic differences between studies. We routinely assess the quality of studies we include in meta-analyses as a potential source of heterogeneity, and a large number of individual quality criteria and quality checklists or scales are available for this purpose [4,5]. Typically, in an individual meta-analysis of randomized controlled trials (RCTs), or in meta-epidemiological datasets that include RCTs from several meta-analyses, each study feature is analyzed as a single dichotomous predictor (trial has the feature or does not) of the effect size of a trial with two arms. For continuous outcomes, to determine whether the study feature is associated with the reported treatment effect size, the difference between the two arms of the RCT is calculated and standardized to estimate an effect size, along with the standard error of the effect size. These measures of effect size are then regressed on the predictor within a meta-analysis framework to see if the trial feature explains some of the variation in effect sizes across RCTs. Sterne et al. [6,7] outline the methodology further, and also apply a similar approach for dichotomous outcomes.

Although a number of study features have been proposed, an actual association with bias in effect size estimates has been empirically confirmed for only a few, and the literature shows conflicting results [8]. Some analyses have demonstrated that low quality trials exaggerate treatment effects [9,10]; for example, low quality trials and those with inadequate treatment allocation concealment showed an increased effect size in a large dataset reported by Moher et al. [11] that included 11 meta-analyses. A summary score consisting of 11 common quality criteria can find absolute differences in effect size of 0.20 between trials meeting a quality threshold and those that do not [12]. Other researchers [13,14] applying quality measures to a number of meta-analyses concluded that individual quality measures were not reliably associated with the strength of treatment effect across studies and clinical areas. Using data from other meta-epidemiological studies [10,11,15,16], Juni et al. [17] reported associations of effect sizes with allocation concealment and double blinding, whereas the generation of treatment allocation did not show a statistically significant effect across datasets. These results highlight an inconsistency between theory and empirical evidence of bias. The question of whether generation of treatment allocation is an important critical appraisal dimension or whether systematic reviewers are wasting time assessing it in systematic reviews is a pertinent one. Thus, authors might ask why they have to adhere to CONSORT reporting guidelines, if empirical evidence is lacking or inconsistent.

Recently, some research has been dedicated to determine variables that may explain the reason for inconsistent results in studies investigating evidence of bias [18]. Wood et al. [19] used three meta-epidemiological datasets to explore the associations of quality features and effect sizes [10,15,20] and investigated whether the nature of the intervention and the type of outcome measure influence the effect of allocation concealment and blinding. They found that trials using subjective outcomes showed exaggerated effect sizes when there was inadequate or unclear allocation concealment or lack of blinding while associations were negligible in trials with objective outcomes. Other factors that are inherent to datasets may influence the ability to detect effects of trial quality or other study moderator effects.

Existing research on the power of meta-analyses to detect study moderator effects in meta-analysis has focused on patient characteristics. Specifically, these papers focus on the decision regarding the appropriateness of meta-regression or individual patient data [21-23]. The meta-regression analyses that we consider in this paper are those in which study level characteristics are of interest; to our knowledge, there has been little direct investigation of the effects of heterogeneity on the power to detect study level moderator effects. Furthermore, simulation has rarely been applied to meta-analytic questions (meta-analyses and meta-regressions pose a complex model with within-trial as well as across-trial variables to consider) but it can be a powerful tool in systematically assessing the effects of hypothesized factors [24-26].

The aim of the study was to determine the power to detect study level moderator effects, such as trial quality, in meta-analysis taking the number of trials, the effects of trial sample size, the quality distribution across trials, and dataset heterogeneity into account.

## Methods

We investigated the effect of factors potentially associated with power in meta-analyses of controlled trials and meta-epidemiological studies to detect a moderator effect via simulation and applied the results to empirical datasets. For this paper, we assumed a dichotomous predictor as the moderating variable, that is, feature present or not (or, for example, ‘high quality’ *versus* ‘low quality’). Furthermore, we selected continuous outcomes for this analysis and used effect size as the measure of treatment effect. The Institutional Review Board HSPC of the RAND Corporation to review research involving human subjects, as required by federal regulations, has reviewed the study and deemed it exempt (ID 2013–0423).

### Simulation design

We used Monte-Carlo simulation to explore the effects of four parameters, systematically varied in the simulations: (1) The number of trials in each meta-analysis was set to 5, 10, 20, 50, 100, or 200 trials. The values were chosen to represent substantial variation in the number of trials found in individual meta-analyses as well as meta-epidemiological studies; (2) The sample size within each trial was set to 20, 50, 100, 200, or 500 participants to represent the substantial variation in the number of participants in existing trials; (3) The moderator effect (that is, the effect of the study-level feature on effect size) was set to 0.0, 0.1, 0.2, 0.3, or 0.4. A moderator effect of 0.2 (for example the effect of trial quality) means the difference in effect sizes between studies with the feature (for example, ‘high quality’ trials) *versus* studies without the feature (for example, ‘low quality’ trials) is 0.2 standard deviations [27]. The value 0.4 represents a very large moderator effect; we are not aware of an empirical study which has detected a moderator effect this large, and additional variation was not considered to be informative; (4) The degree of residual heterogeneity (study variance due to other factors than the studied moderator effect) was quantified using τ^2^, and was set to 0 (no additional heterogeneity apart from that explained by the moderator variable), 0.1, 0.2, 0.4, or 0.8. The values were chosen to represent heterogeneity in individual meta-analyses as well as meta-epidemiological datasets. The indicator τ^2^ represents the amount of heterogeneity in a meta-analysis above that expected by chance. Of note, the heterogeneity measure I^2^, represents 1 minus the proportion of the total variation that is due to chance, thus equal amounts of heterogeneity (τ^2^) give rise to different proportions of variation that is not due to chance (I^2^) [28]. The table in the Additional file 1 shows the relationship between I^2^ and τ^2^ in our simulations. In a meta-analysis which comprised larger individual studies, less variation would be expected by chance; therefore a meta-analysis with larger trials would be expected to have a larger value of I^2^ than a second meta-analysis with smaller trials, while τ^2^ was constant. In addition, we varied the balance of the trial level moderator variable, to either 50% of the trials having the feature, or 25% having the feature. For all modeled variables, values were chosen to represent existing datasets and scenarios encountered by researchers, as well as having substantial variation to aid detection of the effect of the variables.

Allowing each simulation parameter to vary simultaneously produced a total of 6 * 5 * 5 * 5 * 2 = 1,500 simulation possibilities. For each cell of the simulations, we generated and analyzed 1,000 meta-analyses. Given the number of studies per meta-analysis and number of patients per study, the simulations represent 1.5 million meta-analyses, comprising 96.25 million trials and nearly 17 billion simulated patients’ data.

#### Data generation and analysis

Data were generated in two stages using R version 3.02 [29]. In the first instance, we generated a vector for study effect sizes where all trials had an effect size of 0.0. Trials were then assigned to their cell of the simulation, and for each of the cells we added a random variance parameter to increase the heterogeneity between studies to give the appropriate value for τ^2^.

The second stage generated normally distributed data for each trial, with a mean of 0 and a standard deviation of 1, with sample sizes per trial ranging from 20 to 500. The treatment effect for trials with a positive expression of the moderator variable (for example, ‘high quality’ trials) was zero, and the treatment effect of trials with a negative expression of the moderator variable (for example, ‘low quality’ trials) was created by adding a constant (0.0 through 0.4) to the control group of the trials.

Data analysis was carried out using the Metafor package [30]. Effect sizes were calculated using the escalc () function, and meta-regression models were estimated with the rma () function, with DerSimonian-Laird estimation. The categorical moderator effect was used as a predictor in the model. The primary outcome of interest was the statistical significance of the quality moderator effect. For each cell of the simulation we calculated the proportion of analyses in which the result was statistically significant at *P* <0.05.

### Application examples

In addition to the general simulation models we also used the specification of five empirical datasets and modeled results to fit these specific datasets.

First, we obtained a meta-epidemiological dataset by randomly selecting 200 trials from an RCT collection indexed in PubMed and published in 2006 [31] and extracted the primary outcome results for each of the trials. In this meta-epidemiological dataset, some studies had extremely large sample sizes: for the purpose of calculating the mean effect size, these studies were removed from the analysis. The mean sample size in the dataset was 132 and heterogeneity across study results was substantial (I^2^ = 92%, τ^2^ = 0.285). We ran Monte-Carlo simulations to determine the power necessary to detect a moderator effect, such as trial quality. For this moderator effect size power analysis we ran simulations for moderator effects of 0.1 and 0.2, generated 1,000 random effects meta-analyses per simulation, and systematically varied the proportion of studies with the feature (for example, proportion of ‘high quality’ trials).

We also performed post hoc power calculations for four published meta-epidemiologic datasets in a further application. The datasets were assembled to analyze the association of study quality indicators and reported effect sizes. Dataset 1 was derived from all Cochrane Back Review Group reviews of non-surgical treatment for non-specific low back pain in the Cochrane Library 2005, issue 3; the dataset included 216 individual trials [12]. For Dataset 2, prior systematic reviews and meta-analyses conducted by Agency for Healthcare Research and Quality (AHRQ)-funded Evidence-based Practice Centers (EPCs) were searched; this dataset includes 165 trials [8]. Dataset 3 is a replication of a selection of trials used in a published meta-epidemiological study [11] using trials from 11 digestive diseases, mental health, stroke, pregnancy and childbirth, and circulatory disease treatment meta-analyses; this dataset includes 100 trials. Dataset 4 is a replication of parts of another published meta-epidemiological dataset [14] using trials from eight cardiovascular disease and five pediatric treatment meta-analyses; this dataset includes 149 trials. More information on the dataset composition can be found in Hempel et al. [8] and Hempel et al. [18]. The power calculation simulations matched the dataset specifications in terms of heterogeneity (I^2^), number of studies, and mean sample sizes. We computed the power for two representative levels of moderator effects (0.1 and 0.2) and investigated the effect of a potential reduction in the residual heterogeneity (for example, if other sources of heterogeneity were known and could be controlled for).

## Results and discussion

**Figure 1:**
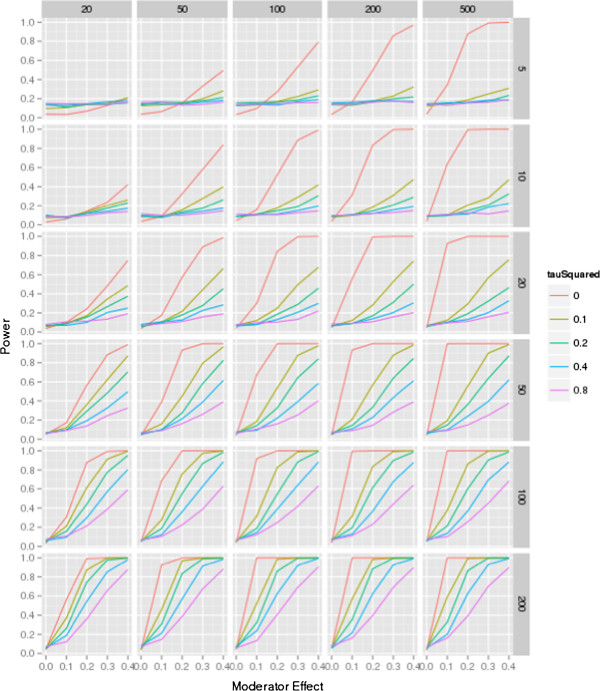
**Power simulation, moderator distribution 50:50.** The figure shows the power for each of the combination (number of studies in each meta-analysis ranging from 5 to 200 controlled trials; study sample size ranging from 20 to 500 participants; moderator effect ranging from 0 to 0.4; residual heterogeneity ranging from τ^2^ = 0 to 0.8; for a 50:50 distribution of the moderator (for example, 50% ‘high quality’, 50% ‘low quality’).

**Figure 2:**
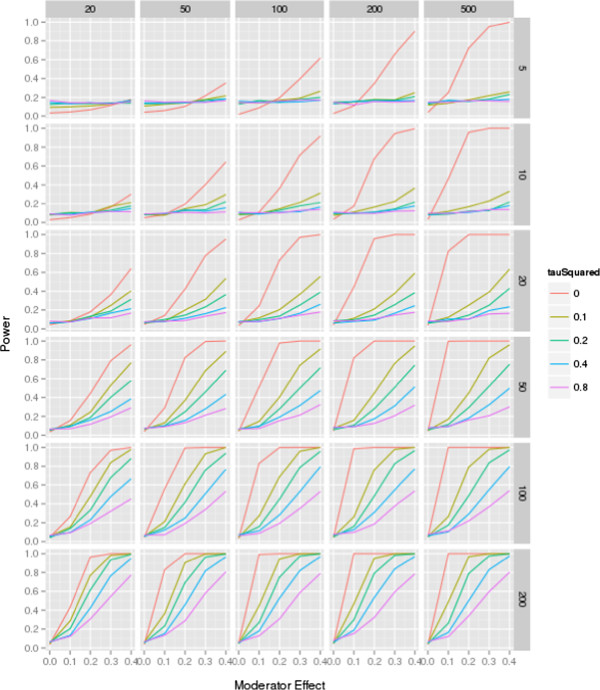
**Power simulation, moderator distribution 25:75 ratio.** The figure shows the power for each of the combination (number of studies in each meta-analysis ranging from 5 to 200 controlled trials; study sample size ranging from 20 to 500 participants; moderator effect ranging from 0 to 0.4; residual heterogeneity ranging from τ^2^ = 0 to 0.8; for a 25:75 distribution of the moderator (for example, 25% ‘high quality’, 75% ‘low quality’).

The results are shown in Figures 1 and 2. Figure 1 is based on meta-analyses that assume an equal distribution of the moderator, for example, a 50:50 ratio of low and high quality studies within the dataset. The figure shows the power for each of the possible meta-analytic conditions for five levels of residual heterogeneity, for six levels of the number of trials per meta-analysis, with five levels of trial sample sizes, and five levels of moderator effects (such as the association of study quality and effect sizes). Both figures may be used as a reference guide for authors to estimate the approximate sample size needed to show a moderator effect.

The first diagram on the top left in the figure shows the power for different levels of residual heterogeneity in a meta-analysis of five trials that each includes 20 participants. There is insufficient power to even detect a large moderator effect (0.4). As the number of participants increases, effects can only be detected in the presence of very little or no residual heterogeneity (that is, noise). With an increasing number of trials, the power to detect a smaller moderator effect (0.1) is <80% in many of the presented scenarios, even in the presence of no residual heterogeneity. However, in a meta-analysis of 100 trials with 500 participants each, detection of substantial moderator effects (0.3 to 0.4) is possible except in the presence of large (for example, 0.4, 0.8) residual heterogeneity.

The figure shows the relative importance of individual variables in the presence of all contributing factors and emerging trends across scenarios. When there is no heterogeneity, the total number of participants in the meta-analysis is the determining factor for power - for example, for a moderator effect of 0.1, approximately 90% power is attained with 100 trials of *n* = 100 (10,000 participants), 50 trials of 200 individuals (also 10,000 participants), and 20 trials of 500 participants (again, 10,000 participants). However, when heterogeneity is present, the number of participants per study is considerably less important than the number of trials - for a moderator effect of 0.2, and τ^2^ of 0.1, 50 trials of 50 participants provides low power - approximately 0.45; increasing the size of the trials to 500 participants increases power by a small amount, to approximately 0.55, despite the increase in the total sample size by a factor of 10; but keeping the size of the trials constant, and doubling the number of trials gives an increase in power to approximately 0.75, and doubling the number of trials again (to 200) increases power to approximately 0.95. This finding is equivalent to the result found in cluster randomized studies, where the number of individuals is less important than the number of clusters [32,33]. The number of studies in the meta-analysis increases the power steadily, even when the sample sizes of the studies and the moderator effect are only small.

The heterogeneity within the dataset is of particular importance for the power. Even when large studies are included in the meta-analyses and a substantial moderator effect is present in the dataset, a large number of studies is needed to detect the effect; when τ^2^ was set to 0.8, a power of 80% was only achieved when investigating an extremely large moderator effect (0.4).

The scenario assumed in Figure 1 (an equal distribution of the moderator variable in the dataset (for example, 50% ‘low quality’ and 50% ‘high quality’ studies)) is rare in empirical meta-analyses. Figure 2 shows a more realistic distribution with only 25% of studies classified as ‘high quality’ and 75% as ‘low quality’ studies. While the trend is similar, in this unbalanced moderator condition, sufficient power to detect a statistically significant moderator effect is achieved in only few scenarios. The analysis shows that a ratio of high and low quality studies moving away from 50%, as is typical for empirical datasets, reduces power further.

### Applications

Before applying critical appraisal criteria and assessing the effects of potential moderators in a meta-regression, researchers should determine how many studies are needed and how much power these studies give to detect a moderator effect, such as the effect of low or high quality in a dataset that may explain variation in treatment estimates across studies. This factor can determine whether the analysis has sufficient power to identify effects, hence it increases our confidence in whether study results are distorted by lack of quality or other study characteristics.

We sampled the described RCT collection [31] to demonstrate the application of the power considerations in an empirical meta-analysis with large heterogeneity (I^2^ = 92%, τ^2^ = 0.285) where researchers are likely to want to investigate possible sources of heterogeneity such as trial quality. We can infer from the simulations that when high heterogeneity is present, the number of RCTs in the dataset is more important than the sample size of the individual trials, and power to detect moderator effects such as the effect of quality on effect sizes is generally low. Table 1 shows the power for moderator effects of 0.1 and 0.2, varying the proportions of studies with a positive expression of the moderator (for example, proportion of ‘high quality’ trials). The table highlights the association of the moderator proportion and the number of RCTs needed to show an effect: As the proportion moves away from 50%, the size of the smaller group matters more (for example, only 20% ‘high quality’ RCTs = 40/200, only 40 RCTs total in one group) than the size of the larger group (for example, even a large number of RCTs with the more common feature). While the difference in power is marginal comparing a ratio of 50% and 30% (power = 75% *versus* 69%, 800 RCTs, moderator effect = 0.1), results are very different for scenarios with only 10% ‘high quality’ and 90% ‘low quality’ RCTs (power = 39%, 800 RCTs, moderator effect = 0.1). When the ratio is far from 50%, the increase in power associated with more RCTs is small. Even in a dataset of 600 RCTs, a power of 80% to show a statistically significant effect is not reached when only a few RCTs show the rare expression of the moderator, for example, only 60 ‘high quality’ RCTs are present (proportion = 0.1).

**Table 1:** Power to determine the number of studies needed to show a moderator effect in a given meta-analysis dataset

	**Proportion of studies (%) with positive expression of moderator (for example, ‘high quality’)**			
**Studies ( *n *)**	**0.5**	**0.3**	**0.2**	**0.1**
**Moderator effect = 0**				
200	6	6	5	6
400	6	5	6	5
600	5	6	6	6
800	6	5	6	7
**Moderator effect = 0.1**				
200	24	22	22	13
400	45	40	33	21
600	65	54	48	29
800	75	69	53	39
**Moderator effect = 0.2**				
200	75	67	57	32
400	95	93	84	60
600	100	99	96	78
800	100	100	98	90

Extrapolated from random sample of 200 RCTs, I^2^ = 92%, τ^2^ = 0.285; mean sample size 132; alpha = 0.05; in the absence of a moderator effect (moderator effect = 0), the power should vary around 5%.

The size of the moderator effect will depend on the individual moderator, for example, the specific quality feature. In practice, it might be possible to modify the criteria to avoid a quality scoring that results in a very imbalanced distribution (which will affect the power negatively as outlined above). However, even if a distribution across studies in equal proportions (50:50) can be reached, simulations show that for a moderator effect of 0.1, the approximate power in the sample of 200 studies is 24%. For a moderator effect of 0.2, the approximate power in the given sample is 75%, that is, still below the common power threshold to justify analyses. Power will be sufficient only for more substantial moderator effects, and judging from the existing critical appraisal literature, effects >0.2 are unusual. Either more RCTs are needed to show an effect, or the residual heterogeneity needs to be reduced by identifying other predictors of effect size differences between studies.

In previous work [8,18] we have described inconsistent results of associations between quality and effect sizes across meta-epidemiological datasets. In post hoc power analyses summarized in Table 2, we observed that conditions have to be ‘just right’ to show a statistically significant moderator effect, even in meta-epidemiological datasets that include a large number of studies. Datasets either featured large heterogeneity thereby minimizing the power to detect even large quality effects (Dataset 2, a collection of EPC evidence reports, τ^2^ = 0.345, 165 studies, I^2^ = 97.5%), or did not show sufficient heterogeneity in effect sizes in the key τ^2^statistic to suggest the need to identify source of variation across studies (τ^2^ = 0.03, 149 studies, I^2^ = 60%; Dataset 4, a replication of parts of a meta-epidemiological dataset by Balk et al. [14]). The post hoc power analyses indicated that sufficient power was present only in two of the four selected datasets (Dataset 1, I^2^ = 72.4%, τ^2^ = 0.305, 216 studies; Dataset 3, I^2^ = 59.6%, τ^2^ = 0.131, 100 studies) and in only one of the datasets were effects of quality shown empirically with a simple regression model as outlined elsewhere [11,12].

**Table 2:** Post hoc power calculations for meta-epidemiological datasets

	**Power for the identified heterogeneity (as present in the empirical dataset)**	**Power in the presence of reduced heterogeneity (if other moderators could be identified and heterogeneity could be reduced)**	**Power in the presence of no residual heterogeneity (if all other moderators could be identified)**
*Dataset 1*			
216 trials, mean sample size 80			
Observed heterogeneity: I^2^ = 72.4%, τ = 0.305			
Modeled residual heterogeneity	14%	0.25%	0%
Moderator effect = 0.1	38%	50%	85%
Moderator effect = 0.2	91%	100%	100%
*Dataset 2*			
165 trials, mean sample size 286			
Observed heterogeneity: I^2^ = 97.5%, τ = 0.345			
Modeled residual heterogeneity	70%	35%	0%
Moderator effect = 0.1	12%	20%	100%
Moderator effect = 0.2	37%	60%	100%
*Dataset 3*			
100 trials, mean sample size 119			
Observed heterogeneity I^2^ = 59.6% τ = 0.131			
Modeled residual heterogeneity	5%	0.25%	0%
Moderator effect = 0.1	42%	58%	73%
Moderator effect = 0.2	92%	99%	100%
*Dataset 4*			
149 trials, mean sample size 342			
Observed heterogeneity I^2^ = %, τ = 0.03			

Dataset characteristics and simulation approach are described in detail elsewhere [18].

### Implications

The simulations show that heterogeneity, in particular, has a distinct effect on the power to detect moderator effects. We have used the example of trial quality to illustrate the effect of a study-level moderator on treatment effect estimates in meta-analyses; however the results are valid for any binary study-level moderator. The implication for meta-analyses and meta-epidemiological dataset analyses are substantial. First, these power considerations may explain the inconsistency in the empirical evidence for bias in medical research. We conclude from the presented data that in order to have sufficient power to discern statistically significant distortion effects of moderators such as trial quality on the true treatment effect (that is, bias), datasets with low residual heterogeneity or very large datasets are needed. A conclusion that a proposed moderator does not have a substantial effect on trial outcomes is prone to a type II error without sufficient power in the dataset being used to test the association. We set up the presented simulations to include moderator effects (the treatment effects in the simulations were modeled to be distorted by the moderator), and we used effects of up to 0.4 in treatment effects, which is a very large effect; however, only under very specific conditions was power sufficient to detect the included moderator effect. Hence, findings such as ‘no effect of quality’ have to be considered in light of the power to detect an effect.

The implications for individual meta-analyses are that current practices for investigating quality and other study characteristics may not be enough to detect study level effects, and statistically insignificant results of meta-regressions should not be interpreted as evidence that characteristics such as study quality do not affect outcomes. The statistical power of individual meta-analyses is lower than the power available in meta-epidemiological studies; however, heterogeneity may be reduced and other assessed variables and potential effect modifiers may be added to the regression model to help isolate effects of study quality. Imbalanced proportions of investigated moderators reduce the power to detect moderator effects. Where possible, criteria should be selected accordingly, particularly for critical appraisal instruments with very strict criteria, such as the Cochrane Risk of Bias tool [34,35], that routinely result in very imbalanced distributions - given that the number of studies with the rare expression of the moderator has pronounced implication for the statistical power and can only be compensated for statistically with a very large number of trials to ensure sufficient power. In situations with greater imbalance or large amounts of heterogeneity, it may be appropriate to relax the conventional 5% alpha cutoff for statistical significance to avoid missing bias.

The nature of meta-epidemiological datasets is that they are diverse and typically cover a number of interventions, clinical indications, and other study features because they are obtained by pooling a number of individual meta-analyses. Consequently, these datasets are typically characterized by considerable heterogeneity. One approach to deal with this characteristic is to statistically control for sources of heterogeneity. Including variables that account for the heterogeneity as covariates can have large effects on power. Each covariate costs (approximately) one degree of freedom, which is (approximately) equal to one study. However, the covariates must not be collinear with each other and must not be collinear with quality features. In addition, systematic factors that account for a substantial amount of noise in the dataset may be difficult to identify. Only a few characteristics, such as the type of outcome measure, seem to be reliably related to the detection of quality effects [19].

An additional issue with the use of power analysis is that an estimate of residual heterogeneity cannot be calculated before data are collected and analyzed; hence power analyses we have described may necessarily be *post hoc*. However, in certain situations an estimate of the residual heterogeneity may be obtainable, for example, when updating a systematic review; hence the power analysis may be carried out *a priori*.

### Limitation

The simulations shown provide a reference guide to researchers when planning meta-regressions. A limitation of simulations is that they must make simplifying assumptions that may reflect the applicability of the results. For example, in these simulations, all trials within a meta-analysis had an equal sample size, while in an empirical dataset, such characteristics would vary, which may alter the power of the analysis. Power analyses are estimates; they estimate the chances of finding a statistically significant result and are therefore only signposts. No other studies have investigated (to our knowledge) the power to detect quality effects (or other study level moderators) under varying levels of study heterogeneity.

## Conclusions

Although study characteristics such as quality may explain some amount of heterogeneity across study results in meta-analyses, the amount of residual heterogeneity in effect sizes is a crucial factor in determining when associations between study features and effect sizes can be statistically detected. Detecting moderator effects requires more statistically powerful analyses than are employed in most published investigations. Hence, negative findings should not be considered as evidence of a lack of effects, and investigations should not be considered hypothesis-proving unless a power calculation shows sufficient ability was demonstrated to detect an effect of a clinically important size, such as 0.10 in treatment effect size differences.

## Competing interests

The authors declare that they have no competing interests.

## Authors’ contributions

SH and JNVM drafted the manuscript. JNVM carried out the power analyses. SH, ZW, and MJB provided data for the underlying model parameter. JNVM, SM, and PS designed the simulation. All authors provided critical revisions and approve the final version of the manuscript.

## Supplementary Material

Additional file 1 **Median value of I**^**2**^**s for cells in simulation.** The table shows the median value of I^2^ for cells in the presented simulations. Click here for file
