# Supplementary material for: Risk of bias: a simulation study of power to detect study-level moderator effects in meta-analysis
Source: Syst Rev. 2013 Nov 28;2:107. doi: 10.1186/2046-4053-2-107 (PMC4219184; doi:10.1186/2046-4053-2-107)
Supplement: Additional file 1 — Median value of I2s for cells in simulation. The table shows the median value of I2 for cells in the presented simulations. [file 2046-4053-2-107-S1.docx]

# Additional file 1: Median Value of I-Squared for Cells in Simulation

Table 1: Median I-squared values

|  |  | **Number of patients** | | | | |
| --- | --- | --- | --- | --- | --- | --- |
|  |  | 20 | 50 | 100 | 200 | 500 |
| **τ^2^** | 0 | 0 | 0 | 0 | 0 | 0 |
|  | 0.1 | 47 | 71 | 83 | 91 | 96 |
|  | 0.2 | 64 | 82 | 91 | 95 | 98 |
|  | 0.4 | 77 | 90 | 95 | 97 | 99 |
|  | 0.8 | 86 | 94 | 97 | 99 | 99 |

Note: Based on simulations generating and analyzing 1,000 meta-analyses. Given equal τ^2^, I^2^ varies as a function of the sample size in the analysis. It does not vary as a function of the number of trials in the analysis. It should be noted that there is not a direct relationship between I^2^ and τ^2^; two meta-analyses may have equal numbers of studies, number of patients per study, and τ^2^, but will not necessarily have the same value for I^2^.
